# A Review on the Utilization of Lignin as a Fermentation Substrate to Produce Lignin-Modifying Enzymes and Other Value-Added Products

**DOI:** 10.3390/molecules26102960

**Published:** 2021-05-16

**Authors:** Attia Iram, Aydin Berenjian, Ali Demirci

**Affiliations:** 1 Department of Agricultural and Biological Engineering, 221 Agricultural Engineering Building, Pennsylvania State University, University Park, PA 16802, USA; axi52@psu.edu (A.I.); amb9476@psu.edu (A.B.); 2 The Huck Institutes of Life Sciences, Pennsylvania State University, University Park, PA 16802, USA

**Keywords:** fermentation, media, lignin-modifying enzymes (LMEs), lignin, microbial substrate, PHAs, microbial lipids

## Abstract

The lignocellulosic biomass is comprised of three major components: cellulose, hemicellulose, and lignin. Among these three, cellulose and hemicellulose were already used for the generation of simple sugars and subsequent value-added products. However, lignin is the least applied material in this regard because of its complex and highly variable nature. Regardless, lignin is the most abundant material, and it can be used to produce value-added products such as lignin-modifying enzymes (LMEs), polyhydroxyalkanoates (PHAs), microbial lipids, vanillin, muconic acid, and many others. This review explores the potential of lignin as the microbial substrate to produce such products. A special focus was given to the different types of lignin and how each one can be used in different microbial and biochemical pathways to produce intermediate products, which can then be used as the value-added products or base to make other products. This review paper will summarize the effectiveness of lignin as a microbial substrate to produce value-added products through microbial fermentations. First, basic structures of lignin along with its types and chemistry are discussed. The subsequent sections highlight LMEs and how such enzymes can enhance the value of lignin by microbial degradation. A major focus was also given to the value-added products that can be produced from lignin.

## 1. Introduction

The massive consumption of fossil fuels and their limited availability presented humankind with the ever-growing problems of decreasing environmental quality and an increasing need for sustainability and energy equality. Therefore, alternative energy resources such as lignocellulosic biomass are being analyzed for their potential to meet the increasing demands of energy stability and equity. Many developed countries are trying to set forth the legislation to utilize lignocellulosic biomass. The U.S. Renewable Fuels Standard (RFS) plans to produce at least 16 billion gallons of biofuels from lignocellulosic biomass by 2022 [1]. On the other hand, European Union is trying to increase the total number of biorefineries. All such efforts entail increases in lignin production, and thus, the need for utilization of lignin in a more environmentally friendly manner [1].

Lignocellulosic biomass is one of the most abundant renewable resources on this planet. The three main components of this biomass are cellulose, hemicellulose, and lignin [2]. Lignin is the second most abundant organic material after cellulose [3]. Currently, 50–70 million metric tons of lignin are produced worldwide every year [4]. The most common sources of industrial lignin are the pulp and paper industries. The renewable fuel standard (RFS) program mandated that at least 60 billion gallons of biofuels should be produced, and this amount requires at least 0.75 billion tons of lignocellulosic biomass. Therefore, the conversion of lignocellulosic biomass will generate at least 0.225 billion tons of lignin as a byproduct [4].

**Figure 1:**
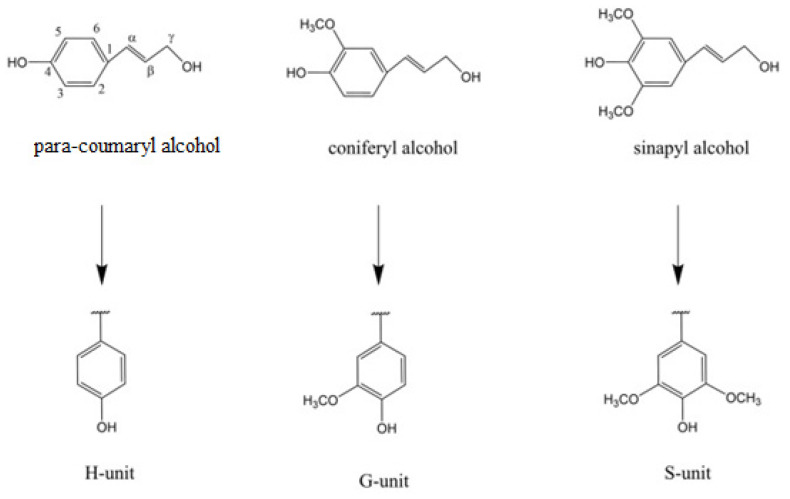
Structure of three lignin monomer alcohols. Numbers represent position of carbon in benzene ring while Greek letters represent distance of carbon atoms in side chain from ring C1 [2].

Lignin provides necessary structural integrity and mechanical strength to the plant [5]; however, this material is also seen as the barrier to the effective usage of lignocellulosic biomass in different industrial sectors. Lignin is made from three different monomer alcohols: coniferyl alcohol, sinapyl alcohol, and coumaryl alcohol (as illustrated in Figure 1). The cellulose and hemicellulose components of lignocellulosic biomass are already in use in value-added products including bioethanol. Cellulose and hemicellulose are polymers of sugar monomers that can be fermented by the microbial species to make bioethanol. However, the natural structure of lignocellulosic biomass is so complex and versatile not only because of the plant species, but also the different formation mechanisms and structural arrangements of cellulose, hemicellulose, and lignin [6,7]. For example, the lignin component in softwood can be up to 25–30% (wt/wt), and it is usually formed by the chemical polymerization and oxidative coupling of coniferyl alcohol, one of the three main building blocks of lignin. On the other hand, hardwood species contain lower amounts (20–25%) of lignin, with variable proportions of both coniferyl alcohol and sinapyl alcohol [6,7].

In most modern refineries, the main desired components are cellulose and hemicellulose, while lignin is considered a byproduct or waste material that ends up as low-quality burning fuel [8]. In the plant cell wall, three components are chemically linked to a complex web in which lignin is predominantly linked to hemicellulose, which is also diverse in its basic chemical composition (e.g., glucomannan, xylan, etc.). Therefore, no simple extraction method is available for the separation of lignin from the other two parts of the cell wall. In modern biorefineries as well as pulp and paper industries, lignin has to be removed from the cellulose and hemicellulose, which is done with different combinations of hydrothermal, chemical, or biological methods [9]. As a result, large quantities of lignin are generated worldwide as a byproduct.

While large quantities of lignin are produced, it is considered a low-value byproduct and is burned to generate heat, which is not the best way nor the most environment-friendly usage of this important organic compound. Currently, various thermochemical techniques are being developed and used at a small scale to generate value-added products from lignin. However, these techniques are not environmentally friendly, and a large amount of energy is needed for the processing of lignin, so these methods are not ideal at an industrial scale. In recent years, a more environment-friendly and effective way of lignin utilization was proposed in the literature. Some examples of such methods are biological or enzymatic conversions of lignin into value-added products [9]. While lignin is complex and diverse in its chemical composition, various microbial species were discovered that can utilize lignin by degrading it into simpler lignin monomers. As a result, various aromatic compounds are formed, which after further microbial processing could be used in the microbial fermentation processes. Therefore, utilization of lignin as a microbial substrate (carbon source) was proposed to increase its overall industrial value.

The lignin-modifying enzymes (LMEs) are the key players in making lignin an effective carbon source for use in fermentation processes. These enzymes not only degrade lignin to various specificity and effectiveness levels, but they can also be produced using lignin as the fermentation media. LMEs also have a large number of applications in various industries. In addition to LMEs, lignin degradation can also result in the production of other value-added products such as microbial lipids, vanillin, and muconic acid. All such products have various applications in food, energy, feed, and pharmaceutical industries. However, the use of lignin as the microbial media is currently in its early stages and should be researched extensively to make it more feasible at an industrial scale.

This review focuses on the basic mechanisms involved in adopting lignin as a microbial feedstock. A special focus was given to the microbial degradation mechanisms of lignin into monomers and the subsequent microbial pathways to breakdown lignin monomers. Current strategies to produce LMEs and other value-added products are also discussed.

## 2. Types of Industrial Lignins and their Production Chemistry

Lignin is the second most abundant organic compound in the world after cellulose [3]. However, unlike cellulose, its production and chemical nature can vary greatly depending on the industrial process used to extract it from the lignocellulosic biomass. Currently, there are several methods to produce lignin from the lignocellulosic biomass, and most are collectively comprised of an industrial process known as pulping. While the three major subtypes of pulping can be categorized into chemical, semimechanical, and mechanical pulping, most of these methods are combined to get a specific type of product [10]. Mechanical pulping is used in the low-cost paper industries. Usually, nonresinous softwoods and some types of hardwoods are used in such industries, and the resultant lignin is yellowish. The mechanical pulping does not involve any type of chemicals other than water or steam. The combination of chemical and mechanical pulping is used for hardwoods that usually give a low-quality pulp. Chemical pulping can create different types of technical lignin such as kraft lignin, soda lignin, and lignosulphonates, which are available in the paper industry in bulk amounts [11]. On the other hand, other lignin types such as hydrolysis lignin, ionic liquid lignin, and organosolv are produced in relatively lower amounts. These processes are summarized as follow:

(i) Kraft Lignin

Kraft lignin is produced from a process known as sulfate or kraft cooking process. In this process, lignin in the wood is dissolved in the aqueous solution of sodium sulfide and sodium hydroxide [11]. As a result, lignin is broken down into fragments of varying sizes, which can then be dissolved into alkaline solutions. The generated solution is a deep brown liquid, which is known as spent liquor. Approximately 80% of the world’s lignin is produced through this method and is known as kraft lignin [11]. However, only a very small proportion (~1–2%) of this lignin is used to make value-added products, while 98–99% is incinerated to produce steam and energy [12,13]. The major portion of it is used to make steam and energy by burning. A distinct feature of kraft lignin’s chemical nature is the presence of a large number of phenolic hydroxyl groups and biphenyl structures [14]. The increasingly condensed structure is dependent on the duration of cooking. The ash content of kraft lignin can be up to 30% if it is not chemically removed from the end-product.

(ii) Soda Lignin

Soda pulping is used for straws and some hardwoods; the pulping is done with soda and anthraquinone [15]. The main difference between soda lignin and kraft lignin is that soda pulping is done in a sulfur-free solution [11]. Anthraquinone is good to decrease carbohydrate degradation [16]. The applications of soda lignin are in phenolic resins, dispersants, and animal nutrients [17]. These applications need the high purity of the lignin while potential toxic compounds in the animal nutrient can be dangerous for animal health [18].

(iii) Lignosulphonates

Lignosulphonates are the byproduct of sulfite cooking and contain a large number of charged particles. The main delignification process is performed with sulfate ions [19]. After sulfonation, lignin is degraded and solubilized [11]. The lignosulphonates have numerous useful chemical compounds, such as carboxylic groups, phenolic hydroxyl groups, and sulfur-containing groups. All of them are good for colloidal and dispersing properties [19].

## 3. Biochemistry of Lignin Degradation

Lignin is a polymer of mainly three aromatic structures: hydroxyphenyl (H), syringyl (S), and guaiacyl (G) [20]. The relative amounts of each of these structures depend on the plant species. The formation starts with the help of an electro-abstracting enzyme such as laccase. The lignin biosynthesis is then achieved through free radical coupling [21]. Just like many other lignocellulosic components, lignin should also be depolymerized before it can be used in various industrial applications. There are two different types of lignin depolymerization techniques. The first and most common in industrial settings is thermochemical depolymerization, which is achieved at high temperatures and chemical additives, catalysts, and other compounds. The second method which is more environmentally friendly and slower than thermochemical process is biological depolymerization. Various microorganisms, mainly fungi and bacteria, are capable of degrading lignin with the help of LMEs [22].

Microbial degradation and conversion of lignin occur through a complex enzymatic system including many different types of enzymes and their intermediate and final products. These enzymes work in synergy to convert lignin into smaller molecules of a different chemical nature. Different types of C–C linkages in the phenylpropane units make lignin difficult to degrade [23]. The source, pulping process, and processing conditions can make lignin different from each other in terms of chemical structure. LMEs act by an oxidative mechanism, not by hydrolytic mechanisms. As such, they were classified into various subclasses based on their specific mechanism of action. Depending on their role in lignin degradation, the ligninolytic enzymes can be divided into two major classes: LMEs and auxiliary enzymes that assist degradation by providing necessary molecules and ions to the LMEs. Some types of LMEs are also known as ligninolytic oxidative enzymes, as they oxidize oxidative cleavage [23]. The enzymes are also differentiated according to their microbial species; both fungal and bacterial lignin-degrading enzymes are identified in the literature [24].

**Figure 2:**
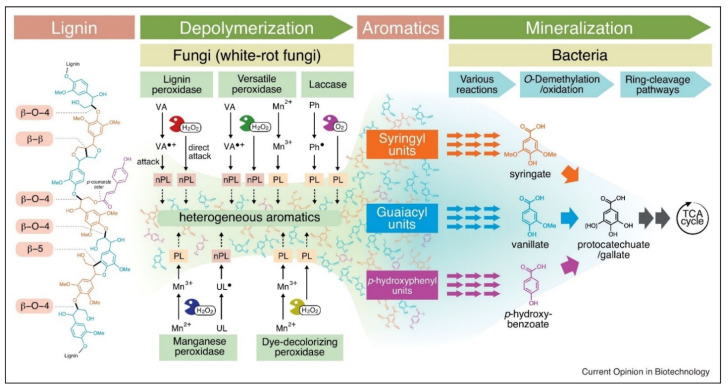
Different enzymatic pathways of microbial lignin degradation [27]. Copyright 2021 by Elsevier.

Various fungal species are best known for producing three different types of heme peroxidases. These are lignin peroxidase (LiP), manganese-dependent peroxidase (MnP), and versatile peroxidase (VP). A detailed description of their mechanism of action with the applications and EC numbers is given in Table 1 and Figure 2. On the other hand, phenol oxidases belong to the multicopper oxidase family, and among them, laccase is primary in terms of industrial applications [25]. The dye-decolorizing peroxidase (DyP) is a new group of heme-containing peroxidases that are present in both fungi and bacteria [23]. These enzymes are different from other heme peroxidases such as LiP, MnP, and VP. There are many different types of auxiliary enzymes that help the heme peroxidases and laccases in the complete degradation of lignin. These are known as oxidoreductases, and some examples include glyoxal oxidase, aryl alcohol oxidase (veratryl alcohol oxidase), pyranose 2-oxidase (glucose 1-oxidase), and cellobiose/quinone oxidoreductase [26].

**Table 1:** Lignin-modifying enzymes (LMEs) and their applications.

Enzyme Class	Mechanism of Action	EC Number	Applications	References
Lignin peroxidases (LiPs)	H_2_O_2_-dependent oxidative depolymerization of lignin	EC 1.11.1.14	Delignification in pulp industry, bioleaching, etc.	[28]
Manganese peroxidases (MnPs)	Oxidation of one electron donor Mn2+ to Mn3+ and subsequent oxidation of phenolic substrates	EC 1.11.1.13	Treatment of dye wastewater	[24]
Versatile peroxidases (VPs)	Combined oxidation-active sites	EC 1.11.1.16	In combination with LiPs and MnPs	[24,29]
Laccases	multicopper oxidases with one electron oxidation	EC 1.10.3.2	Food, paper, and textile industries	[24,30]
Heme peroxidases (DyPs)	Heme peroxidases under low pH	EC 1.11.1.19	Degradation of anthraquinone dyes in textile industry	[24,31]
Oxidoreductases	glyoxal oxidase aryl alcohol oxidase pyranose 2-oxidase and many others	EC 1.2.3.5, EC 1.1.3.7, EC 1.1.3.4, etc.	In combination with other enzymes	[24,28]

Bacterial lignin-degrading enzymes are not studied extensively compared to that of fungal enzymes. However, many different bacterial strains with the ability to produce LMEs were isolated from soil and other natural habitats. Among the various identified LMEs, LiPs and DyPs are reported extensively in the literature [27]. Most of the studies describe different homologs of DyPs that were found in the bacterial strains with the ability to degrade lignin in a different capacity compared to that of fungal strains. Bacterial species are usually slower at degrading lignin than fungal species. Bacterial laccases are another set of enzymes with the capacity to effectively degrade nonphenolic substrates [32]. Among various strategies to use bacterial laccases in the detoxification of dyes, the genes are cloned and overexpressed in various research setups [22,23]. The research on the isolation and characterization of different bacterial species, which can produce a high amount of LMEs, has been conducted and published in the last decade [33,34].

In the last two decades, there was evidence that fungal strains, especially white and brown rot fungi, can degrade plant cell wall components through the production of free hydroxyl ions (OH^−^). Firstly, hydrogen peroxide (H_2_O_2_) is produced, which assists with oxidization and production of OH^-^. These free radicals then attack different cell wall components along with lignin and cleave the bonds between these structures. As a result, the plant cell wall becomes more susceptible to the action of LMEs and other lignocellulolytic enzymes (as illustrated in Figure 2) [35]. There are three different pathways through which fungi create hydroxyl ions. These are Fenton reactions catalyzed by glycopeptides, quinone redox cycling, and cellobiose dehydrogenase catalyzed reactions [36].

Cellobiose dehydrogenase, or CDH, is a monomeric enzyme that helps in the oxidation of oligosaccharides in the plant cellulose and hemicellulose, thus helping the enzymes that degrade these two components. CDH helps with the production of H_2_O_2_ in the Fenton reactions, which then help the heme peroxidases to degrade lignin. CDH is known to take part in the depolymerization of all three plant cell wall components. Low molecular weight peptides are also produced by some fungal species, such as *Gloeophyllum trabeum*, which can help with cellulose degradation into smaller molecules by a set of oxidative reactions, such as quinones generation [37]. Similarly, glycopeptides were also found in white-rot fungi, and they assist lignin degradation by using hydroxyl ions. All these oxidative reactions create a complex cascade that ultimately helps the lignin-degrading enzymes to penetrate plant cell walls more effectively and with the hydrolysis of lignocellulosic structures [38].

The lignin degradation pathways described above are usually done by fungal and bacterial species in the natural environment where these species use plant cell wall martial for growth and survival. However, these pathways can be manipulated and adopted on an industrial scale to produce LMEs and other value-added products from technical lignin (byproducts of pulping, etc.). The microbial fermentation process after microbial lignin degradation can be used to enhance the production of such value-added products. Many research articles were published with the possibility of using biological lignin degradation pathways to produce value-added products from lignin, and thus, enhance the industrial value of lignin byproducts. Such literature reports are described in subsequent sections of this review.

## 4. Biochemical Pathways to Produce Lignin Value-Added Products

**Figure 3:**
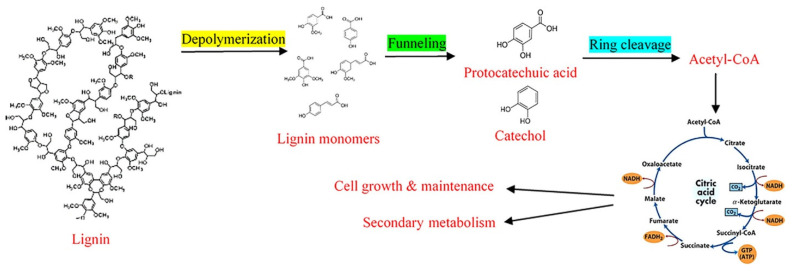
Production of value-added products from lignin after microbial degradation [9]. Copyright 2021 by John Wiley and Sons.

After lignin biodegradation through enzymatic and nonenzymatic pathways, the microbes are left with lignin-derived aromatic compounds of a variable polymerization degree and length along with lignin monomers. These aromatic compounds are detrimental for most living species and cannot be used for growth or any other metabolic process. However, a few microbial species can further change these aromatic compounds into intermediary products, such as protocatechuic acid and catechol, which can then be further processed by the microbial species (as illustrated in Figure 3). After processing these intermediary compounds, the simple carbon compounds can then enter the metabolic and growth cycle of microbes. These chemicals are then used for cell growth and product synthesis. Lignin monomers after biodegradation can enter into the microbial pathway through two common steps: biological funneling and ring cleavage [9]. After ring cleavage, the resultant compounds can be taken up by the common metabolic pathways, such as the citric acid cycle or Krebs cycle (as illustrated in Figure 3) [9].

Microbial conversion of lignin monomers into value-added products and growth is mostly studied in bacterial species such as *Pseudomonas putida* and *Sphingomonas paucimobilis* [39,40]. In the biological funneling, the lignin monomers are converted into certain types of intermediary compounds which are phenolic in nature, and this could be the rate-limiting step in the lignin conversion into value-added products. Biological funneling is a complex set of different pathways involving many different enzymes, such as acryl-CoA hydratase, acryl-CoA synthetase, dehydrogenase, O-demethylase, and decarboxylases [41]. The nature and number of such pathways depend on the microorganisms and their environment. Under aerobic conditions, the commonly created intermediate product is protocatechuate [39,40]. Different pathways such as the CoA-dependent β-oxidation pathway, CoA-independent pathway, and the CoA-dependent non-β-oxidation pathway were associated with the aerobic conversion of H-type lignin monomers, such as 4-coumarate. Firstly, the H-type compounds are converted into 4-hydroxybenzoate, which are then converted into protocatechuate with the help of 4-hydroxybenzoate-3-hydroxylase, and all these steps use oxygen as the oxidant [39,40,42]. For G-type compounds, similar pathways are identified (as illustrated in Figure 2 and Figure 3). However, the intermediate product is vanillic acid instead of 4-hydroxybenzoate. In this set of biological funneling, vanillate demethylase is the key enzyme. The intermediate product is protocatechuate, which is then ready to go to the next set of microbial conversion pathways. The conversion of S-type molecules is more complex as it has two distinct methoxy groups. The end-product in this case is 3-O-methylgallate, or it can be further converted into gallate. The intermediate product is syringate or 3-O-methylgallate depending on the type of the reaction, and the key enzyme is tetrahydrofolate-dependent O-demethylase. The end-products can then enter into the second step of conversion which is the ring cleavage pathways [42].

The most common and basic strategy for ring cleavage in an aerobic environment is with oxygenases. These enzymes use activated molecular oxygen to hydroxylate the aromatic ring [42]. The first set of aerobic pathways utilize ring-cleaving dioxygenases for the conversion of funneling products into compounds that can be taken up by the cellular metabolic pathways. The cleavage can be between the two hydroxyl groups (ortho position) or adjacent to the hydroxyl groups (meta position). The end-product in most of these pathways is β-ketoadipate [42]. In the ortho-cleavage pathways, two enzymes (protocatechuate 3,4-dioxygenase and catechol 1,2-dioxygenase) are involved. These enzymes incorporate both oxygen atoms into the ring-cleavage product. On the other hand, the enzymes involved in the meta-cleavage are known as extradiol dioxygenases that utilize nonheme Fe II or some other divalent metal ions to cleave aromatic rings [42]. The lactone is another intermediate product in these pathways which is also converted into β-ketoadipate. The intermediate products can be further modified into the products of the tricarboxylic acid cycle (TCA). The overall equation of this aerobic pathway is as follows:

Benzoate + 2O_2_ + H_2_O + HS-CoA → acetyl-CoA + succinate + CO_2_ + H^+^ (1)

A detailed description and step-by-step guide of this pathway is given by Fuchs et al. [42].

The second set of enzymes in the aerobic ring cleavage depends on the activities of monooxygenases. While this strategy is in aerobic microbes, it is adopted for the low or fluctuating oxygen environments. In such pathways, phenylacetyl-CoA and Benzoyl-CoA can be used in aerobic or anaerobic pathways depending on the availability of oxygen. Monooxygenases are a family of the class I di-iron proteins that have a diverse di-iron binding site. These enzymes are also known as epoxidases which create a form of nonaromatic epoxides of phenylacetyl-CoA and Benzoyl-CoA. The end reaction in this pathway is the hydrolysis resulting in ring cleavage [42]. In the anaerobic or low-energy fermentations, the ring cleavage pathways are adopted to use none or lesser amounts of ATP. The key enzyme in such reactions could be benzoyl-CoA reductase, which is used in a process known as electron bifurcation [43]. Various types of intermediates and end-products are formed as the result of both aerobic and anaerobic ring cleavage pathways which are then taken by cellular anabolic pathways for growth and product accumulation [9].

The products of ring-cleavage enter the central metabolism pathways such as the citric acid cycle with the help of CoA [44]. At this stage, the fate of the ring-cleavage products depends on the microbial species that are utilizing such compounds [45,46]. For example, in the case of the oleaginous species, acetyl-CoA is used to produce fatty acids with the help of lysases [47]. PHAs can be produced with the help of another set of enzymes (known as PHA synthase) and intermediate products [48]. On the other hand, products such as vanillin or muconic acid are produced from completely different or interrelated pathways [49,50,51]. While vanillin can be produced with ferulic acid biotransformation, muconic acid can be produced with intermediates from glucose metabolism, such as 3-dehydroshikimate [9].

The complexity of microbial systems for the production of such metabolic products is being analyzed through various genomic and metabolic engineering techniques [52]. However, further research is needed to adapt these systems to industrial scales to produce such compounds from lignin byproducts. Strategies to produce such compounds could be developed through genetic engineering or culture optimization techniques. Many research articles were published recently focusing on the potential of different microbial species that are involved in lignin degradation or using aromatic compounds for their metabolic and energy needs [53,54]. The utilization of such a process at industrial scales is difficult, but it could be done through further investigation.

## 5. LMEs and Their Production Using Lignin

While the isolation and identification of microbial species that can produce LMEs were researched extensively in recent years, there were also reports about cultivating known and unknown microbial species in different nutrient and culture conditions to enhance the natural production of LMEs. Published research from the last five years on this area is summarized in Table 2. As can be seen in this table, various carbon sources are analyzed for their efficiency in producing different types of LMEs. Among them, glucose is the most common one as it is the simple sugar that can play a key role in biomass growth [37,38]. While many other carbon sources can be applied for microbial growth, the carbon source can vary greatly in effectiveness if the main goal is to produce a specific kind of enzyme such as LMEs. For that purpose, it is necessary to know the overall effectiveness of the carbon source along with the carbon-to-nitrogen ratio [55]. The most commonly explored nitrogen sources for the production of LMEs are ammonium tartrate, ammonium sulfate, peptone, and yeast extract [55,56,57,58].

The basic purpose of ideal carbon and nitrogen sources with their appropriate ratio is to achieve the minimum growth level, after which the microbial species tend to survive by producing the required enzymes [62]. Therefore, the carbon and nitrogen sources should be limiting nutrients. This induces the secondary metabolic state in the microbial culture. As a result of the nutritional shortage in the culture, a scarcity of amino acids is induced. This results in the production of transfer ribonucleic acids (tRNAs), which induce the secondary metabolic genes [63]. All these steps are carried out in very delicate cellular signaling cascades, and therefore, the determination of ideal carbon and nitrogen sources can be difficult. Various statistical optimization techniques such as response surface methodology were developed to discover the ideal concentrations of such elements [64,65]. Other than carbon and nitrogen, other nutrient elements such as trace elements, organic acids, and their relative ratios are also important to maximize the production of LMEs [66].

Among various other carbon sources, lignin holds a special place because of its diverse chemical moieties that can induce LME production [67]. Lignin comprises 20–40% of lignocellulosic biomass, and this part can be used as the enrichment feedstock and sole carbon source in the production of LMEs by bacterial and fungal species [67]. In this regard, several research articles were published in the last two decades with a special focus on natural lignin, such as chestnut shells [68], wood chips [69], citrus waste [64], and many others. Natural lignin sources also have other plant materials such as cellulose, hemicellulose, pectin, starch, and many others [64]. The pretreatment strategies of such biomass can yield simple sugars that can be used by the microbial species for growth [70]. The focus of such a research project is to develop some pretreatment strategies to make not only LMEs, but also other lignocellulolytic enzymes such as cellulases and hemicellulases [71].

While the focus of many research setups in the past was to establish pretreatment and fermentation strategies for producing LMEs, there is an increased focus on the synthetic or industrial lignins such as kraft lignin and alkaline lignin. Some entries in Table 2 represent alkaline lignin as the carbon source for the production of LMEs. Many other research setups were established in the past two decades to focus on the same concept. Currently, kraft lignin was also used as the carbon source for screening LME-producing bacteria from soil and other natural sources [67]. However, it was also established that industrial lignin, which is the byproduct of pulp and paper industries and is present abundantly, might not be able to provide an effective microbial substrate without some preprocessing techniques.

Therefore, what can be done to the commercial lignins to make them more accessible to microbial species in culture media? Lignin valorization is one technique in this regard. It was established to produce various other microbial products such as microbial lipids [72]. However, commercial lignins as the carbon source for producing LMEs were not explored extensively, and there is much more potential in making this byproduct more valuable. The type of lignin under study also plays an important role, as it will determine what type of fermentation and microbial species can be used. For example, for alkali lignins, the high pH of the media along with microbial species who thrive in the high pH should be selected, or the pH of the media can be adjusted after adding the alkali lignin. However, this step might change the chemistry of the lignin, making it inaccessible for microbial degradation. Some examples of such research setups are the articles reported by Sahadevan et al. [56] and Yang et al. [58]. The majority of research setups, however, deal with lower pH, as it is favorable for fungal enzyme production [73].

In conclusion, LMEs have many applications in the current textile, paper, and food industries. The demand for LMEs is increasing as more and more lignin is being produced by different pulping processes. These LMEs can help in the degradation of lignin to make more valuable products, which will be discussed in the following sections. The usage of commercial lignin to identify and isolate LME-producing microbial species was reported in the literature. Commercial lignins can also be used as the carbon source to produce such enzymes. However, more research is needed on this topic along with the processes to make lignin more accessible for microbial usage. Currently, natural lignin sources such as wood chips are analyzed for their potential to make LMEs. The natural lignin sources are more feasible in terms of microbial culture conditions, such as temperature and pH. However, commercial lignins can also be used as the fermentation media for microbial strains which can adapt to the commercial lignin environment.

## 6. Other Value-Added Products Using Lignin as the Microbial Carbon Source

Besides LMEs, many other microbial products were proposed to increase the value of industrial lignin byproducts. Some examples of such products include polyhydroxyalkanoates (PHAs), microbial lipids, vanillin, and muconic acids among many others. While such products are useful in different industries and applications, their production from technical lignins can further enhance the value of lignocellulosic biomass at industrial scales.

One of the most commonly converted products from lignin is microbial lipids, which are produced by oleaginous species [74]. Oleaginous microbial species are microorganisms that have a lipid content of more than 20% of their percent dry weight [75]. Almost all of the microbial families have species that can produce lipids or oil content. For example, in the case of bacteria, prominent species with lipid-producing capacities are *Acinetobacter calcoaceticus* (lipid content: 27–38%), *Bacillus alcalophilus* (18–24%), and *Rhodococcus opacus* (~25%). In the case of yeast, *Candida curvata* (58%), *Lipomyces starkeyi* (64%), and *Rhodotorula glutinis* (72%) are the most prominent ones. Fungal species such as *Aspergillus oryzae* (57%), *Humicola lanuginose* (75%), and *Mortierella isabelline* (86%) can also produce high amounts of lipids, as shown according to their percent dry weights. On the other hand, many microalgal species were reported to have lipid production ranging from 16–77% based on the species and environment [74].

*Rhodococcus* species, such as *R. opacus,* were reported for the production of microbial lipids by using lignin as the microbial substrates [76]. While such species can grow on different types of aromatic compounds, their ability to degrade commercial lignins is dependent on various factors including the carbon/nitrogen ratios and culture conditions along with the mineral composition of the media. The enhancement techniques can increase the production of microbial lipids. In a study, 20% of the dry weight of *R. opacus* was reported when grown on lignin base compounds such as vanillic acid and hydroxybenzoic acid [77]. In another study, the same strain of *R. opacus* (DSM 1069) was used to analyze the lipid production trends using kraft lignin as the base media. However, the strain showed poor performance in terms of lipid production before any kind of pretreatment. After reporting these results, the authors introduced the concept of oxygen pretreatment, which is a form of oxygen delignification process under alkaline conditions and is used to obtain smaller molecular weights of lignin [78]. After the oxygen pretreatment, the same strain increased lipid production [78]. Among other types of lignins, alkaline lignin and organosolv were also explored for their potential as the carbon source for producing microbial lipids. In other fermentation enhancement techniques, glucose was used in addition to pretreated lignin to enhance growth and lipid production [79]. Just like LMEs, the carbon-to-nitrogen ratio is another critical factor in the effective production of value-added moieties.

PHAs are known as degradable plastics, which are the polyesters of hydroxyalkanoic acid (HA). They are usually produced as the secondary metabolic pathways in response to stress. PHAs are produced by a wide range of both Gram-positive and negative bacterial species [80]. PHAs can be elastomeric polyesters or thermoplastic esters of HA. In such a mechanism, microbial species use these compounds as energy storage compounds. Structurally, these compounds are classified according to the number of carbon atoms, which can range from 4–14, and the nature of monomer chains: homopolymers or heteropolymers. Both plants and microbial species can produce PHAs; however, microbial species are prominent in the industrial production of such chemicals [81]. While the production of PHAs can be triggered by many different types of simple molecules, the ability of microorganisms to both degrade and then produce PHAs from the degradation products is rare in the microbial world. Among different bacterial species, *P. putida* is one of the species that can degrade lignin as well as produce PHAs [82]. Different strains can behave differently according to different lignin model compounds. The major difference is in the biological funneling process, which is the rate-limiting step in the conversion of lignin monomers into the metabolic compounds. Recently, more studies were conducted to quantify the production of microbial PHAs along with the degradation of lignin [83]. In all such studies, the basic goal was to analyze the effect of different lignin degradation pathways on the production of various types of PHAs. For example, in the case of *P. putida*, it was determined that the basic lignin degradation pathway is based on DyP enzyme systems, while a variety of catabolic pathways were used for funneling and ring cleavage. The research on the enhanced production of PHAs using microbial species can decrease the dependence on mineral-based plastics. Therefore, it is imperative that more research projects with different culture strategies should be conceptualized.

While microbial lipids and PHAs are better known for their ability to replace fossil fuels in the energy sector, there are many other value-added products that can be formed by using lignin as the microbial substrate. Two prominent examples in this regard are vanillin and muconic acid. Vanillin is mainly used in cleaning products, flavoring agents, and perfumes [84]. Recently, this compound also gained interest in pharmaceuticals and cosmetics [85]. While vanillin is usually produced through a chemical synthesis process, recent developments in the biological production of vanillin using lignin as the biorenewable substrate gained interest. There are two different ways through which vanillin can be made by microorganisms using lignin. In the first method, vanillin can be extracted as the intermediate product of biological lignin degradation. The second method utilizes the ferulic acid transformation pathway [86]. Different genetic engineering techniques are being used to utilize the microbial capability of producing vanillin from lignin [87].

Muconic acid and other dicarboxylic acids are some other products of lignin bioconversion into value-added products. Such acids can be used to make the compounds like nylon, polyethylene terephthalate (PET), and polyurethane [88]. Just like vanillin, muconic acid can also be produced as the intermediate product during the microbial degradation of the lignin, or it can be produced as the intermediate product of glucose metabolism through genetic engineering techniques. Several studies were reported with different microbial strains such as *Aspergillus spp* or *P. putida* to produce muconic after genetic modification [50,89]. In conclusion, it can be assumed that microbial lignin degradation can become a platform to produce such dicarboxylic acids if there is an increased research focus on this topic.

## 7. Challenges in the Utilization of Lignin as the Microbial Carbon Source

Lignin is one of the most abundant compounds on earth, and its utilization through environmentally friendly techniques can help solve many problems associated with the energy sector [90]. However, lignin is also one of the most complex materials, and its degradation presents various challenges at an industrial scale [91]. The resistance of lignin to microbial degradation is the main reason it is not being used even after its large production as the byproduct in pulp and paper industries. The utilization of lignocellulosic biomass to make fuels further enhances the need for lignin utilization in effective ways [9]. Microbial lignin degradation is problematic because of its low efficiency and rate. One of the main problems in this regard is the higher range of specificity of lignin-degrading microbes. For example, one microbial species is specialized in breaking only one or a few types of bonds, while the broader range of molecules remain unaffected in the lignin media [78]. This is why commercial lignins are not yet adopted for use in the fermentation media as the carbon source.

Another problem in using lignin as the fermentation media arises again from its complex structure and the presence of different chemical molecules [91]. Many lignin degradation products can be toxic to living organisms, including some microbial strains [92]. The microorganisms that can breakdown lignin and use its monomers in their growth cycle adapted the complex metabolic pathways that can switch from one enzymatic reaction to another based on the chemical structure of their substrate [9]. These pathways work in the order of lignin degradation, funneling, ring cleavage, and metabolic cascade reactions. Along with growth, microbial species also use these pathways for stress regulation and bioproduct synthesis. The problem with using commercial pulping lignin in the fermentation media is that its structure is very different from the natural lignin present in plant species [91]. As a result, there could be a long lag phase or total inhibition of cells due to the production of toxic aromatic compounds, which inhibits some microbial strains.

Another problem in the adoption of lignin in the fermentation media are limitations in genetic engineering and metabolic enhancement techniques [93]. While genetic engineering techniques are mostly developed for bacterial species, these species are not ideal enzyme producers in the case of lignocellulolytic enzymes. Fungal species are reported in the literature for the production of the vast array of such enzymes along with their industrial production [35]. However, fungal strains cannot be genetically engineered as easily as bacterial strains. Similarly, in the case of culture enhancements and media optimization techniques, fungal species present problems in the adaption of submerged fermentation techniques which can easily be scaled up compared to that of solid-state fermentation [94,95]. All such problems in the techniques make the lignin degradation methods by microbes difficult. Additionally, more research is needed to improve such techniques along with the development of new techniques that easily control the production of aromatic compounds in lignin degradation.

## 8. Methods to Enhance the Production of Value-Added Products from Lignin

Lignin degradation and uptake of resultant aromatic compounds by microbial species can be enhanced by applying more diverse and controlled lignin pretreatment techniques. These methods can be both biological and/or thermochemical. Application of thermochemical treatment of lignin before using it in the fermentation media can enhance the degree of depolymerization, which in turn can help the lignin degraders to break smaller lignin chains into monomers. However, effective control of parameters such as temperature and concentration of chemicals is extremely important, as the severe treatment methods can generate many toxic compounds in the lignin slurry. The biological treatments can comprise individual enzymes of their mixtures which can degrade lignin at much lower temperatures compared to that of thermochemical treatment methods [96].

Understanding all lignin degradation pathways could help in controlling such mechanisms, and thus, decrease the possibility of producing toxic intermediate products [92]. As mentioned earlier, the current genetic engineering methods are mostly applied to bacterial strains only, as their growth conditions are easy to control. The same goes for the understanding of molecular pathways involved in lignin biodegradation. Most of the mechanisms are studied through the genetic modeling techniques of bacterial species while much is yet to be discovered in the case of other microbial species, such as filamentous fungi. More research should focus on the understanding of microbial degradation.

The culture enhancement techniques and media optimization are some other techniques that can be used for the production of lignin-derived microbial products [72]. For example, glucose can be added to the media at the initial stages of the incubation to enhance the growth before microorganisms can start degrading the lignin in the media for their energy needs. In addition, the control of dissolved oxygen (DO) and pH can also play important roles in the bioconversion of lignin [72]. Such strategies increased lipid concentration in the fed-batch reactor from 0.3–0.5 g/L [72]. The secondary metabolites can be produced by adjusting the limiting elements in the media [79]. Carbon-to-nitrogen ratios were also studied and proven to play a critical role in lignin depolymerization and production of value-added products [92]. Genetic and metabolic engineering techniques were used many times in the literature reports to enhance the production of value-added products in the last few decades. New microbial consortia are being discovered from soil and other natural environments that can degrade lignin effectively. The newly discovered species should also be analyzed for their potential to produce lignin-derived products at industrial scales. In conclusion, all such strategies should be explored more extensively to know the effect of culture enhancement techniques for lignin bioconversion and production of value-added products from lignin.

## 9. Concluding Remarks and Future Trends

Lignin is one of the most abundant and complex compounds on earth. Its production in the traditional pulping processes and new lignocellulolytic biorefineries increased dramatically in the last few decades. However, it is still being used as a low-value byproduct and is usually burned to generate heat. The effective degradation and subsequent use in the microbial pathways can increase its value, and thus, solve many problems associated with the energy sectors. LMEs can be used to degrade lignin for microbial accessibility. Lignin can also be used to produce such enzymes through microbial fermentation. Other products such as muconic acid, PHAs, and microbial lipids can also be produced from various microbial species. The research on the production of such value-added products is currently very limited and more studies are needed to further understand the underlying mechanisms of microbial degradation of lignin. This would help with the development of industrial processes for the usage of lignin as the microbial substrate.

## Figures and Tables

**Table 2:** Culture enhancement strategies to produce LMEs.

Enzyme System	Growth Conditions/Media	Microbial Species/ Consortia	Enzyme Reaction Rate	Reference
LiP and MnP	Glucose = 10 g/L as carbon source, Ammonium tartrate (Variable concentrations) as Nitrogen source, pH = 4.4 and Temp = 30 °C	*Phanerochaete chrysosporium*	LiP = 150 U/mL, MnP = 0.73 U/ml	[55]
LiP, MnP, and Lac	Glucose = 1%, Lignin = 0.1%, peptone = 0.5%, PH = 9, Temp = Ambient	Uncultured alkalophilic dimorphic lignin-degrading Deuteromycete	LiP = 5.27 U/mg, MnP = 13.18 U/mg, Lac = 4.77 U/mg	[56]
LiP, MnP and Lac	Alkali lignin = 1 g/L, (NH_4_)_2_SO_4_ = 2 g/L, Agitation = 220 rpm, Temp = 29 °C	*Myrothecium verrucaria*	LiP = 0.78 U/g, MnP = 1.31 U/g, Lac = 6.61 U/g	[57]
LiP, MnP, and Lac	Alkali lignin = 5 g/L, yeast extract = 0.1 g/L, pH = 7, Temp = 37 °C	Burkholderia sp. H1	LiP = 2.00 U/L, MnP = 1.12 U/L, Lac = 2 · 25 U/L,	[58]
MnP	Lignin = 100 mg/L, pH = 7.6, Temp = 32 °C, Agitation = 120 rpm	*Bacillus aryabhattai*	MnP = 4.7 IU/mL	[59]
versatile peroxidase (rVP1)	Glucose = 20 g/L, Yeast extract = 5 g/L, Peptone = 5 g/L, Agitation = 150 rpm, Temp = 28 °C	*Physisporinus vitreus* PF18	rVP1 = 23.1 U/mg	[60]
LiP, MnP, Lac, VP-Mn, and VP-Ind	Glucose = 0.5%, Yeast extract = 0.2%, pH = 4.5, Temp = 25 °C	Fungal consortia	LiP = 0.05 ukat/L, MnP = 7.8 ukat/L, Lac = 1.3 ukat/L, VP-Mn = 0.15 ukat/L, VP-Ind = 0.23 ukat/L	[61]
